# Supplementary material for: A living biobank of canine mammary tumor organoids as a comparative model for human breast cancer
Source: Sci Rep. 2022 Oct 27;12:18051. doi: 10.1038/s41598-022-21706-2 (PMC9614008; doi:10.1038/s41598-022-21706-2)
Supplement: Supplementary file 1 — Supplementary Information 1. [file 41598_2022_21706_MOESM1_ESM.docx]

**A living biobank of canine mammary tumor organoids as a comparative model for human breast cancer**

Marine Inglebert^1,2^, Martina Dettwiler^1,3^, Kerstin Hahn^1^, Anna Letko^4^, Cord Drögemüller^4^, John Doench^5^, Adam Brown^5^, Yasin Memari^6^, Helen Davies^6^, Andrea Degasperi^6^, Serena Nik-Zainal^6^, Sven Rottenberg^1,7,8*^

**Supplementary information**

**Figure S1. Hormone receptor status of CMT organoids following cryopreservation or extended passaging**

**Figure S2.** Comparison between unknown dog signature and SBS57 PCR-related sequencing artefact

**Figure S3.** Conservation of the genetic landscape of CMT in patient-derived ORG

**Figure S4.** CMT organoids allow *in vitr*o drug testing

**Table S1.** Clinical information of all patients

**Table S2.** Clinical information and characterization of patients presenting malignant tumors

**Table S3.** Detailed characterization of tissue/organoid pairs

**Table S4.** Estimation of the number of mutations associated with each Single Base Substitution Signatures (SBS) following signature fit approach and comparison of prevalence of SBS between CMT and HBC

**Table S5.** Genes targeted by the custom canine CRISPR library and gRNAs counts (plasmid DNA and day 0)

**Table S6.** Canine mammary tumor organoid medium components

**Table S7.** Primary antibodies used for immunohistochemistry

**Table S8.** Primers sequences used for Sanger sequencing and TIDE analysis

**Table S9.** Primers sequences used for gene editing

**
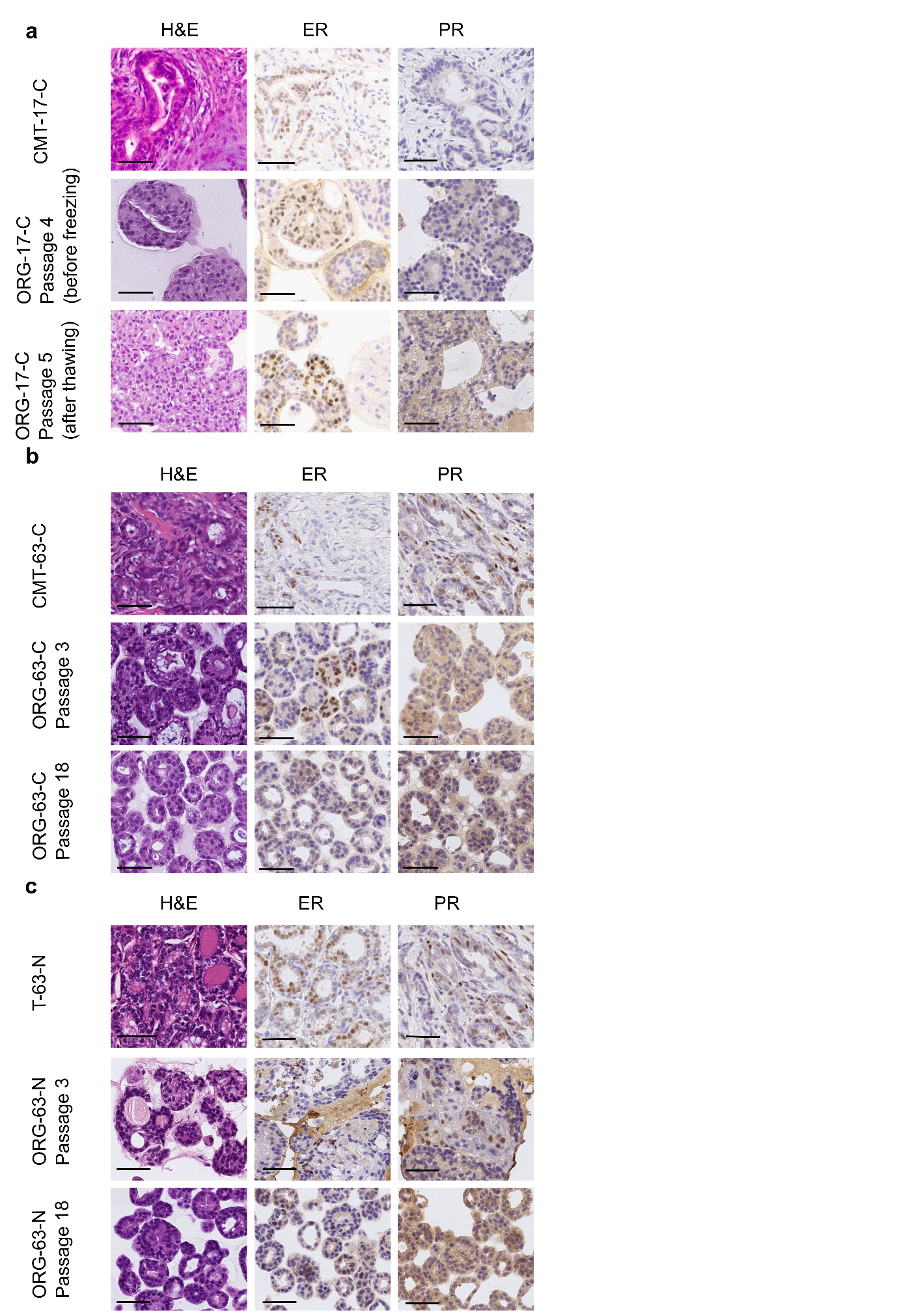
**

**Figure S1. Hormone receptor status of CMT organoids following cryopreservation or extended passaging**

1. Representative images of H&E staining and immunohistochemical analyses of estrogen receptor (ER) and progesterone receptor (PR) in CMT-17-C and tumor-derived organoids ORG-17-C (early passages before and after cryopreservation). Scale bar, 50 μm.
2. Representative images of H&E staining and immunohistochemical analyses of estrogen receptor (ER) and progesterone receptor (PR) in CMT-63-C and tumor-derived organoids ORG-63-C (early and later passages). Scale bar, 50 μm.
3. Representative images of H&E staining and immunohistochemical analyses of estrogen receptor (ER) and progesterone receptor (PR) in normal mammary epithelium (T-63-N) and tissue-derived organoids ORG-63-N (early and later passages). Scale bar, 50 μm.

**Figure S2. Comparison between unknown dog signature and SBS57 PCR-related sequencing artefact**

The main difference between the two signatures seems to be the much higher frequency of T>A mutations in the TTA context in the dog signature.

**
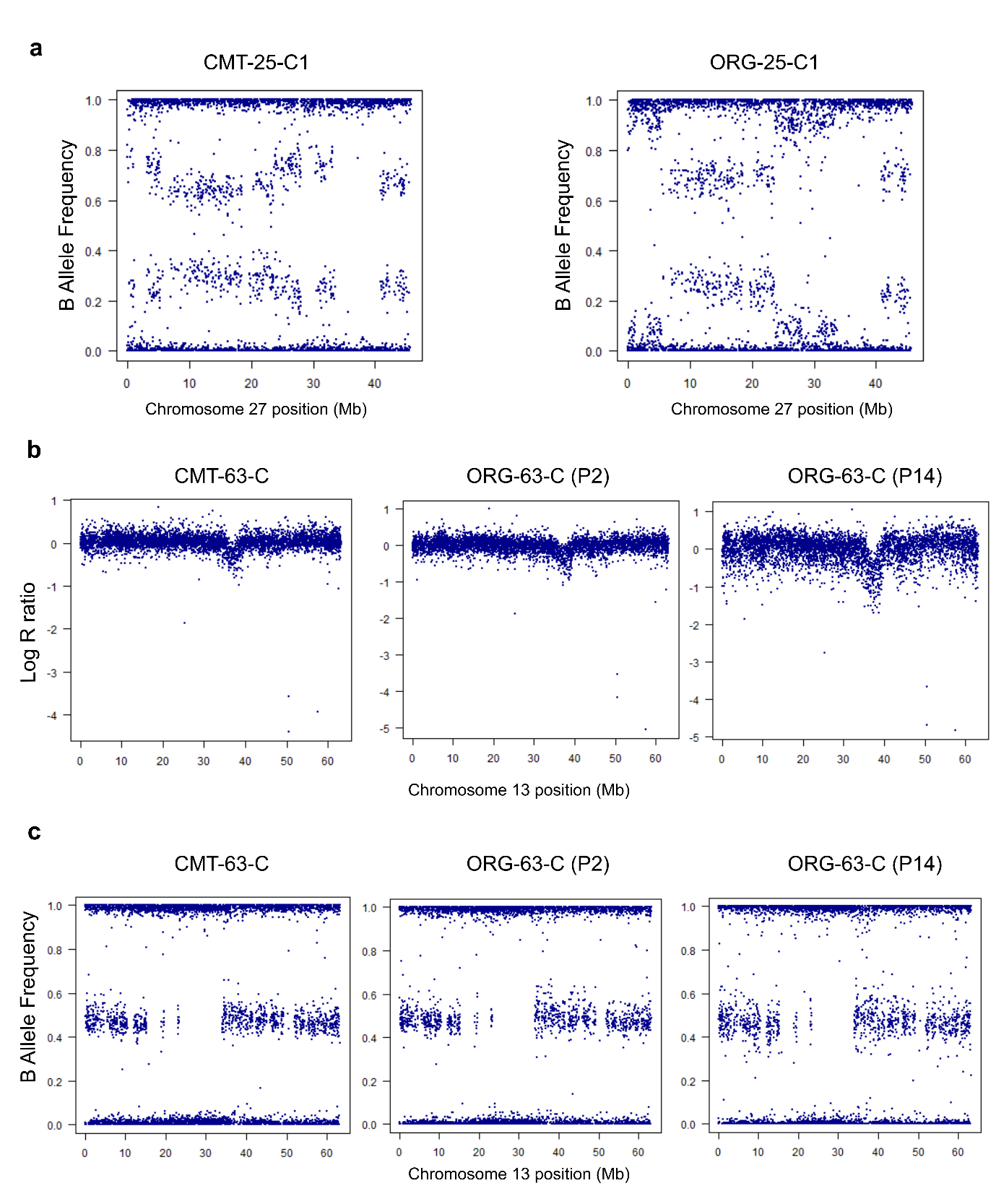
**


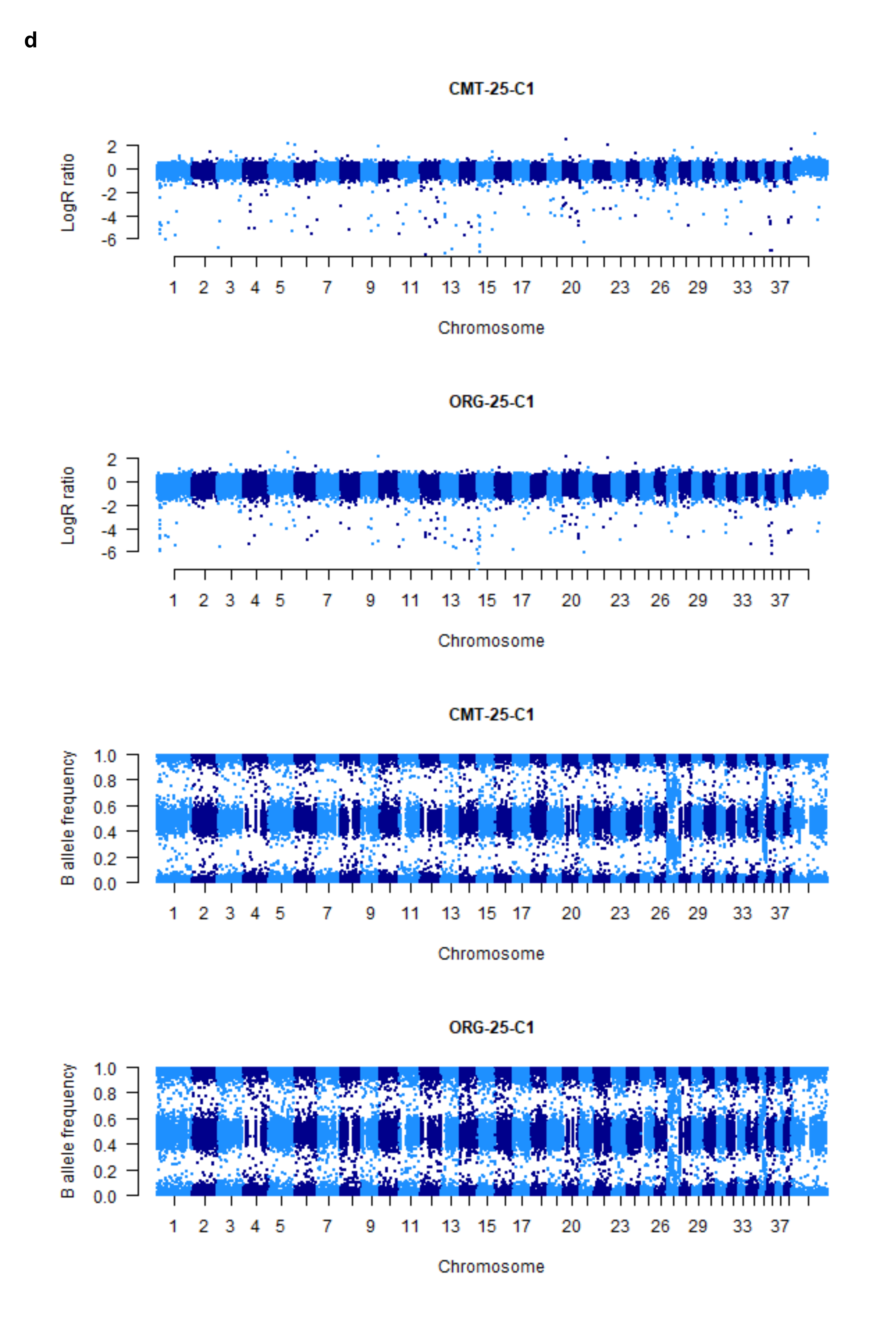

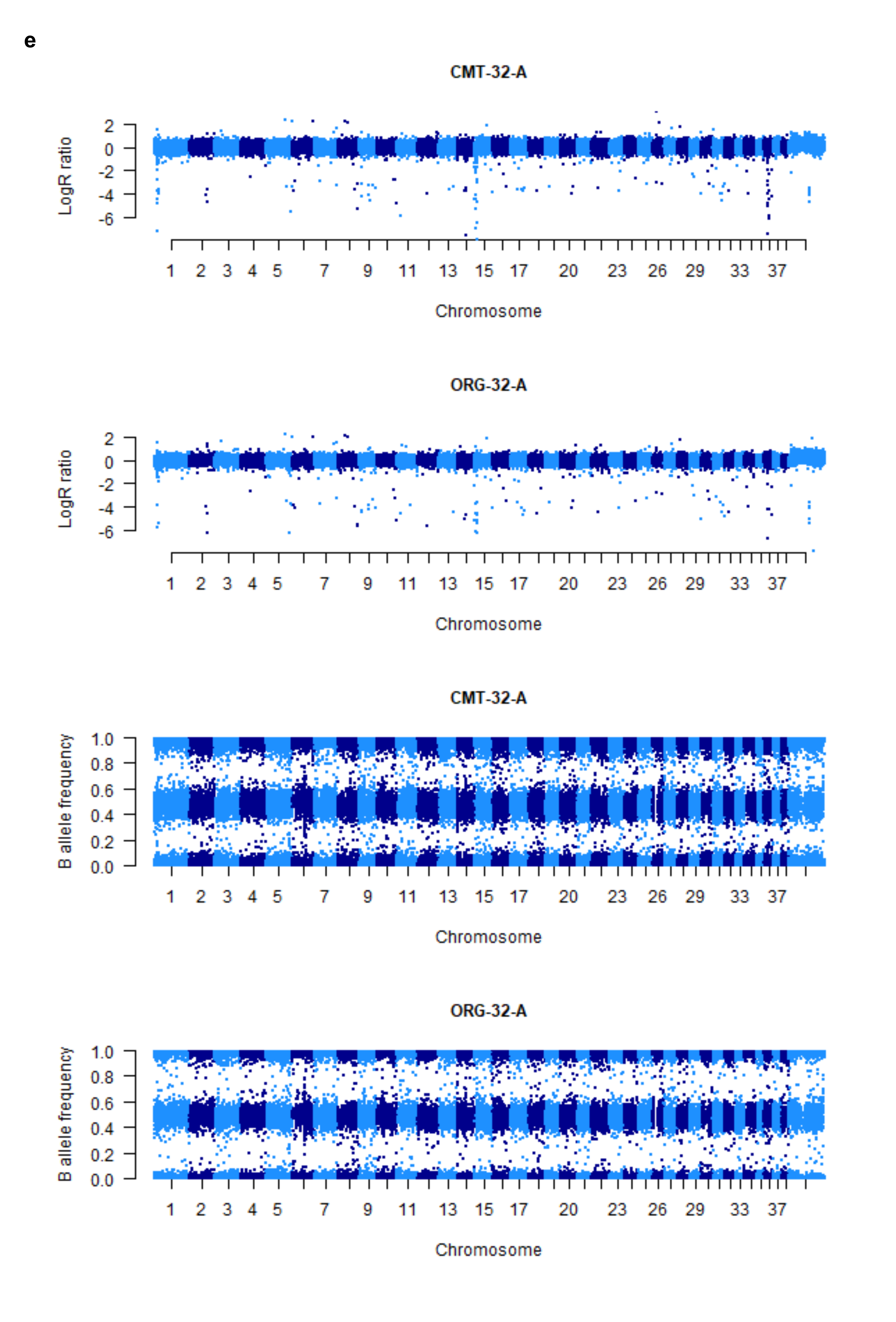

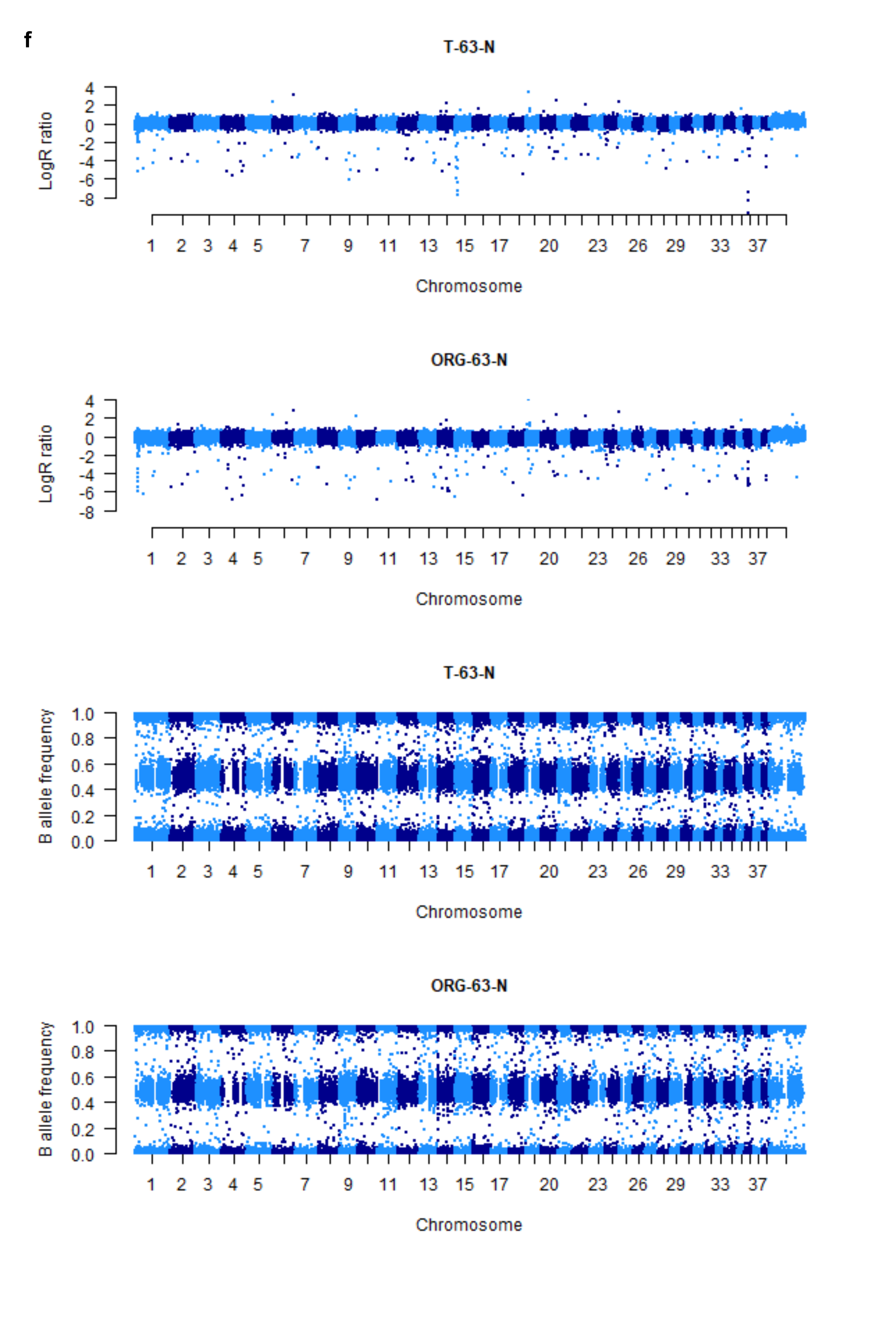

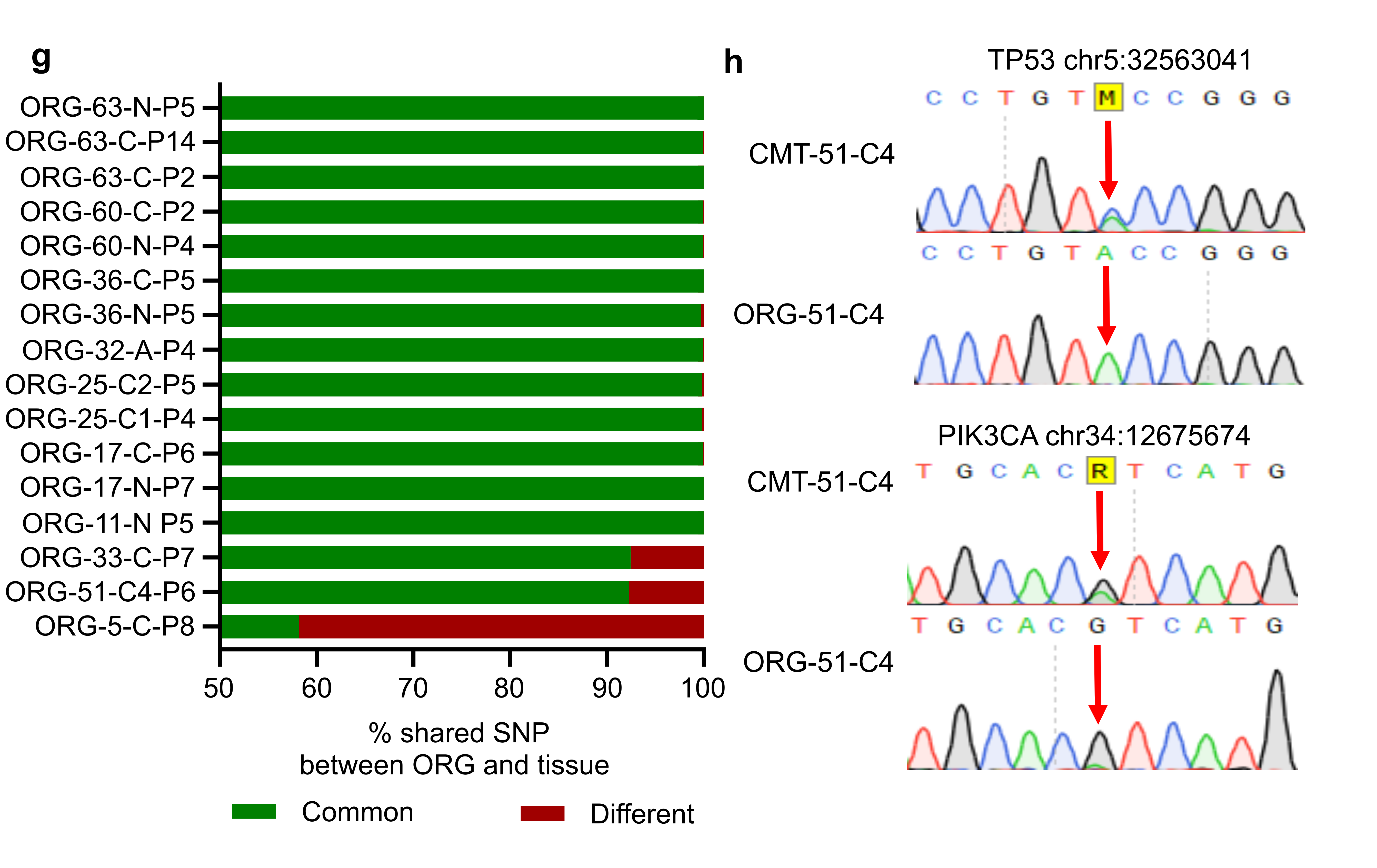


**Figure S3. Conservation of the genetic landscape of CMT in patient-derived ORG**

1. B-allele frequencies on chromosome 27 for matched pair CMT-25-C1 / ORG-25-C1. See also Figure 3B.
2. Log R ratios on chromosome 13 for matched pair CMT-63-C / ORG-63-C (P2) and later passage (P) ORG-63-C (P14). Note the conservation of the SNP distribution in the organoid line after extended time in culture.
3. B-allele frequencies on chromosome 13 for matched pair CMT-63-C / ORG-63-C (P2) and later passage ORG-63-C (P14). Note the conservation of the SNP distribution in the organoid line after extended time in culture.
4. Genome-wide SNP distribution of log R ratios on top part, B-allele frequencies on the bottom part for carcinoma-organoid pair CMT-25-C1 / ORG-25-C1.
5. Genome-wide SNP distribution of log R ratios on top part, B-allele frequencies on the bottom part for adenoma-organoid pair CMT-32-A / ORG-32-A.
6. Genome-wide SNP distribution of log R ratios on top part, B-allele frequencies on the bottom part for non-neoplastic mammary tissue-organoid pair T-63-N / ORG-63-N.
7. Proportion of shared SNP genotypes between primary tissues and matched organoids.
8. Chromatograms of point mutations for matched pair CMT-51-C4 / ORG-51-C4. Top panel: *TP53* chr5:32563041, bottom panel: *PIK3CA* chr34:12675674


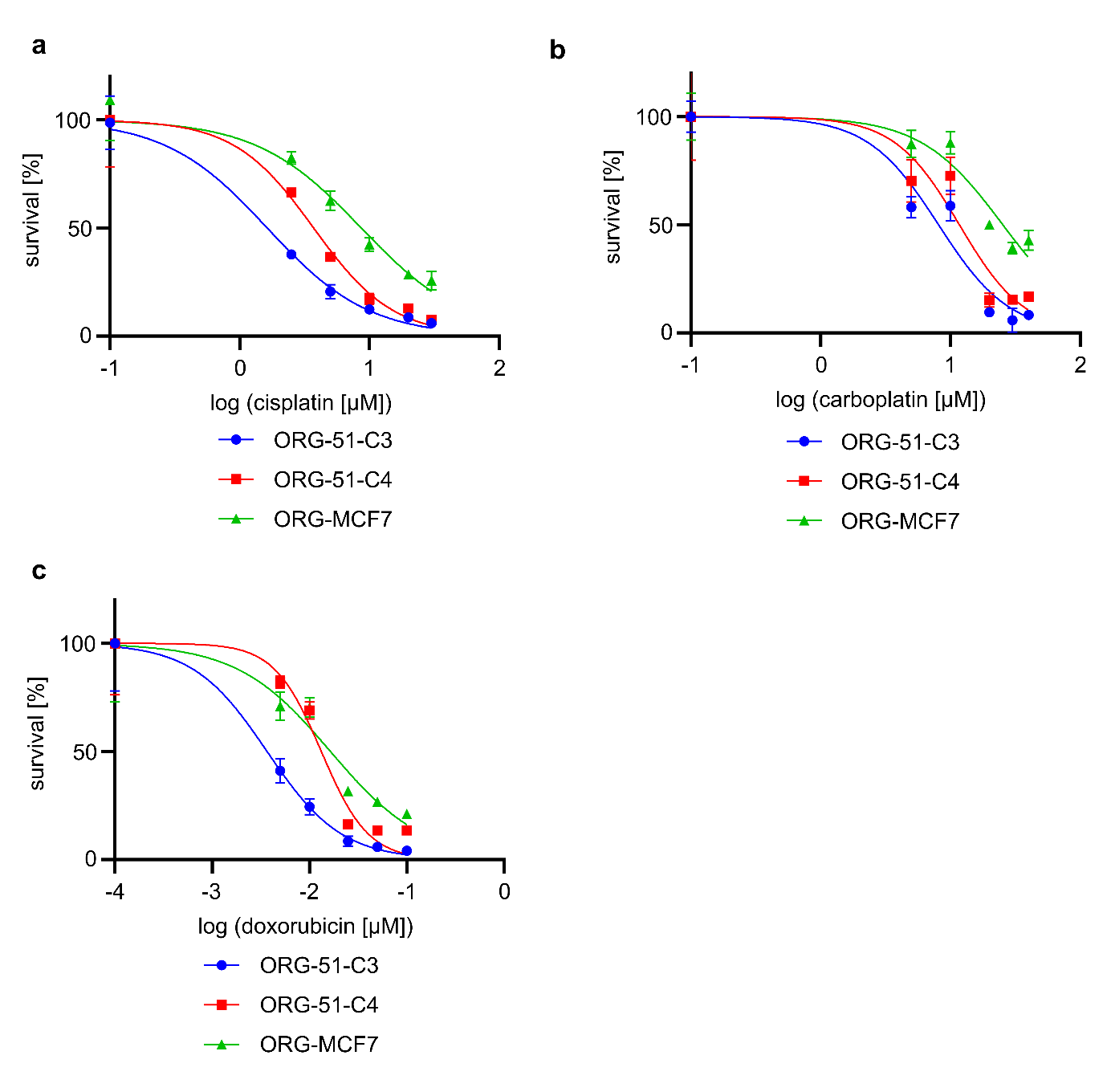


**Figure S4. CMT organoids allow *in vitr*o drug testing**

1. Dose-response curves indicating viability 8 days after treatment with cisplatin. Error bars represent SD of three independent experiments.
2. Dose-response curves indicating viability 8 days after treatment with carboplatin. Error bars represent SD of three independent experiments.
3. Dose-response curves indicating viability 8 days after treatment with doxorubicin. Error bars represent SD of three independent experiments.

**Table S6.** Canine mammary tumor organoid medium components

|  | Medium components | Company | Catalog number | Final concentration |
| --- | --- | --- | --- | --- |
| ADDF+++ | GlutaMax 100x | GIBCO | 35050-038 | 1x |
|  | Hepes 1M | GIBCO | 15630-056 | 10 mM |
|  | Penicillin/Streptomycin | GIBCO | 15070-063 | 50 U·ml-1 |
|  | Primocin | Invivogen | Ant-pm-1 | 50 mg·ml^-1^ |
|  | Advanced DMEM/F12 | GIBCO | 12634-010 | 1x |
| Growth factors | R-Spondin 3 (RSPO3-Fc Fusion Protein) conditioned medium | U protein express | R001 | 10% |
|  | Noggin-Fc fusion Protein conditioned medium | U protein express | N002 | 2% |
|  | Neuregulin 1 | Peprotech | 100-03 | 5 nM |
|  | FGF 7 | Peprotech | 100-19 | 5 ng·ml^-1^ |
|  | FGF 10 | Peprotech | 100-26 | 20 ng·ml^-1^ |
|  | EGF | Peprotech | AF-100-15 | 0.5 ng·ml^-1^ |
|  | A83-01 | Tocris | 2939 | 500 nM |
|  | Y-27632 | Abmole | Y-27632 | 5 mM |
|  | SB202190 | Sigma | S7067 | 1 mM |
|  | B27 supplement | Gibco | 17504-44 | 1x |
|  | N-Acetylcysteine | Sigma | A9165-5g | 1.25 mM |
|  | Nicotinamide | Sigma | N0636 | 10 mM |

**Table S7.** Primary antibodies used for immunohistochemistry

| Antibody against | Clone | Source^1)^ | Antibody Dilution  Antibody Concentration^2)^ | Pretreatment of slides^3)^ | ^4)^ |
| --- | --- | --- | --- | --- | --- |
| Cytokeratin 5/6 (CK5/6) | D5/16B4 | Dako (Agilent) | 1:100 0.51 µg/ml | H2(30) | B |
| CAM5.2 (CK7/8) | CAM 5.2 | BD Biosciences | 1:150 0.505 µg/ml | H2(20)95 | B |
| Cytokeratin 14 (CK14) | LL002 | Leica Novocastra | 1:150 0.16 µg/ml | H2(40)95 | B |
| p63 | 4A4 | Biocare Medical | 1:150 | H2(30) | B |
| MIB-1 (Ki-67 Antigen) | MIB-1 | Dako (Agilent) | 1:50 0.92 µg/ml | H2(30) | B |
| Estrogen Receptor (ER) | EP1 | Dako (Agilent) | 1:50 3.00 µg/ml | H2(40) | B |
| Progesteron Receptor (PR) | polyclonal | Abcam | 1:100 | H2(30) | B |
| Pathway HER2/neu (Her2) | 4B5 | Ventana (Roche) | RTU 6 µg/ml | CC1(36) | V |
| Vimentin | V9 | Dako (Agilent) | 1:1000 0.32 µg/ml | H2(10)95 | B |

^1)^ Abcam, Cambridge, UK; BD Biosciences, San Jose CA, USA; Biocare Medical, Pacheco CA, USA; Dako, Glostrup, Denmark; Leica Biosystems (Novocastra), Newcastle-upon-Tyne, UK; Ventana Medical Systems (Roche Diagnostics), Tucson AZ, USA.

^2)^ Dilutions and working concentrations are given where known; dilutions only are given for antibodies where the Ig concentration is not specified by the manufacturer. RTU = Ready-to-use antibody.

^3)^ H2(30): Pretreatment with Epitope Retrieval Buffer Type 2 (Tris-EDTA, pH 9) (Leica Biosystems) for 30 minutes at 100°C on Bond-III immunostainers. H2(40): idem, 40 minutes at 100°C. H2(20)95: idem, 20 minutes at 95°C. H2(40)95: idem, 40 minutes at 95°C. H2(10)95: idem, 10 minutes at 95°C.

CC1(36): Pretreatment with Ultra Cell Conditioning Solution CC1 (Tris) (Roche) for (36) minutes at 95°C on Ventana BenchMark ULTRA immunostainers

^4)^ Immunostainer used: B = Bond-III (Leica Biosystems); V = Benchmark Ultra (Ventana Medical Systems).

**Table S8.** Primers sequences used for sanger sequencing and TIDE analysis, and screen analysis

| Gene | guide | Primer | Sequence (5´-3´) | Tm(°C) for PCR protocol (*) |
| --- | --- | --- | --- | --- |
| VIM | sgRNA1 | Forward | ACCATGTCCACCAGGTCTGT | 63°C |
|  |  | Reverse | CGGAGGAGCGCGTGGCGTAC |  |
|  | sgRNA2 | Forward | GCTCACCAATTTTTGGCACT | 63°C |
|  |  | Reverse | GAAAAGTGCTGGTGCTCACA |  |
| AKT1 | | Forward | CTGTCCCCTCGATGCCAC | 64°C |
|  |  | Reverse | GCCACGGAGAAGTTGTTGAG |  |
| PIK3CA | | Forward | CCCATTTGCTCCATACTGATCA | 63°C |
|  |  | Reverse | CATGCTGCTTAATGGTGTGGA |  |
| KRAS | | Forward | TCGTCACTGAATTTTCTGAAGCA | 63°C |
|  |  | Reverse | TGGGCCTGCACAAATCAATA |  |
| TP53 | | Forward | TTCCCATCCATTCCTGTCCC | 64°C |
|  |  | Reverse | CCCTCCTTCACCTCCTCTTG |  |
| P5 primer: AATGATACGGCGACCACCGAGATCTACACTCTTTCCCTACACGACGCTCT TCCGATCT[s] TTGTGGAAAGGACGAAAC*A*C*C*G, where [s] is the barcode region. | | | | |
| P7 primer:  CAAGCAGAAGACGGCATACGAGAT [s] GTGACTGGAGTTCAGAC  GTGTGCTCTTCCGATCTCCAATTCCCACTCCTTTCAAG*A*C*C*T, where [s] is the barcode region. | | | | |

(*) Target loci were amplified following a 3-step protocol: (a) 98°C for 30 s, (b) 35 cycles at 98°C for 10 s, Tm(°C) for 20 s, and 72°C for 20 s, (c) 72°C for 5 min.

**Table S9.** Primers sequences used for gene editing

| Gene | guide | Sequence (5´-3´) | Exon targeted |
| --- | --- | --- | --- |
| Vimentin | sgRNA1 | GTACGCCACGCGCTCCTCCG | 1 |
|  | sgRNA2 | AGACACTATTGGCCGCCTGC | 6 |
| Non-targeting gRNA | | GTGATTGGGGGTCGTTCGCCA | No gene targeted |
